# Influence of an immunoglobulin-enriched (IgG, IgA, IgM) solution on activation and immunomodulatory functions of peripheral blood mononuclear cells in a LPS second-hit model

**DOI:** 10.1186/cc9664

**Published:** 2011-03-11

**Authors:** C Duerr, A De Martin, M Sachet, T Konrad, S Baumann, A Spittler

**Affiliations:** 1 Medical University of Vienna, Austria

## Introduction

Immunoglobulin molecules have opposing functions by inducing proinflammatory and anti-inflammatory responses in innate immune effector cells. In the setting of acute inflammation, Toll-like receptors sense the presence of microbial components within minutes. TLR signalling in monocytes and macrophages leads to the production of numerous proinflammatory cytokines which accumulate in the activation of both the innate and adaptive immune system. It is well established that repeated endotoxin stimulation triggers immunological hyporesponsiveness of the monocytic lineage, which is demonstrated by a reduced capacity to produce TNFα upon LPS stimulation. In an *in vitro *model we investigated the impact of immunoglobulins on activation of mononuclear cells obtained from healthy probands and from patients suffering Gram-negative sepsis.

## Methods

Whole blood (*n *= 5) and PBMCs (*n *= 5) from healthy volunteers as well as whole blood from patients in the early (*n *= 8) and in the late (*n *= 8) phase of sepsis were treated with an immunoglobulin-enriched solution containing IgG, IgA, and IgM (IgGAM). Cells were challenged with various concentrations of LPS in a second-hit model and TNFα secretion was measured by ELISA. In addition, monocyte HLA-DR, CD64 and CD11b expression as well as phagocytosis and oxidative burst were analysed by flow cytometry. Proliferation and cytokine release of ConA and/or IL-2 stimulated lymphocytes were undertaken.

## Results

In healthy donors upon two-time LPS stimulation IgGAM incubation resulted in a significant decrease of TNFα secretion administration in a time-dependent and dose-dependent manner. Similar effects were observed in whole blood from patients in the early phase of sepsis. HLA-DR, CD11b and CD64 expression from monocytes of healthy probands declined significantly after LPS expression, which was not observed in septic patients. Interestingly in both groups the administration of IgGAM had no effects on phagocytosis and oxidative burst. Lymphocyte proliferation and cytokine release were significantly impaired in both groups.

## Conclusions

The immunoglobulin-enriched solution possesses a distinct immune modulatory effect *in vitro *on monocytes/monocyte-derived macrophages and lymphocytes from both septic patients and healthy volunteers, especially upon short-term LPS exposure and in the early phase of sepsis.

